# A Novel Docetaxel-Biotin Chemical Conjugate for Prostate Cancer Treatment

**DOI:** 10.3390/molecules27030961

**Published:** 2022-01-31

**Authors:** Mahmoud Rayan, Seba Shadafny, Adam Falah, Mizied Falah, Saleh Abu-Lafi, Sare Asli, Anwar Rayan

**Affiliations:** 1 Drug Discovery Informatics Lab, QRC-Qasemi Research Center, Al-Qasemi Academic College, Baka EL-Garbiah 30100, Israel; mahmoud_ryan@hotmail.com; 2 R&D Department, IDD Therapeutics LTD, Nazareth 1711102, Israel; sshdafny@yahoo.com; 3 Chemistry Education Branch, Iksal Comprehensive School, Iksal 16920, Israel; 4 Science Department, York University, Toronto, ON M3J 1P3, Canada; adamfalah7@gmail.com; 5 Institute for Medical Research, Holy Family Hospital, Nazareth 16000, Israel; mizied.falah@gmail.com; 6 Faculty of Pharmacy, Al-Quds University, Abu-Dies 144, Palestine; sabulafi@staff.alquds.edu; 7 The Institute of Applied Research, Galilee Society, Shefa-Amr 2020, Israel; sareasli@gmail.com; 8 Faculty of Science, Al-Qasemi Academic College, Baka EL-Garbiah 30100, Israel

**Keywords:** taxanes, docetaxel, paclitaxel, biotin, cancer chemotherapy, prostate cancer, formulation

## Abstract

A novel conjugate of docetaxel and biotin (designated as IDD-1010) was designed and chemically synthesized via an ester linkage at position 2’ carbon in docetaxel. The synthesized pure IDD-1010 exhibits a potent anti-cancer activity in in vitro and in vivo studies. At 10 nM, IDD-1010 has induced increased apoptosis and mitotic arrest of PC3-Luc prostate cancer cells, causing aneuploidy and cell death at higher concentrations. Toxicology studies indicate that the maximal tolerated dose (MTD) of IDD-1010 is 150 mg/kg in mice; equivalent to about 12.2 mg/kg of body weight, or to about an 850 mg dose for a patient weighing 70 kg. The MTD-treated mice exhibited weight gain similar to that of the control group, with no gross pathological signs at 14 days post-dosing. At a lower dose, IDD-1010 treatment did not lead to any significant weight loss in mice, although decreased the tumor volume stemming from injecting cancer cells into the dorsal loop of mouse prostate, and it was found to be more potent than Paclitaxel (reference drug). Similarly, IDD-1010 treatment significantly reduced tumor weight and thereby increased the percentage of mice survival as compared to reference drug-treated and control groups. To summarize, the described experiments using IDD-1010, as compared to the reference drug, strongly suggest a potential treatment utility with a wider therapeutic window for prostate cancer. Henceforth, clinical research on such a novel drug candidate would be greatly worthwhile.

## 1. Introduction

**Figure 1:**
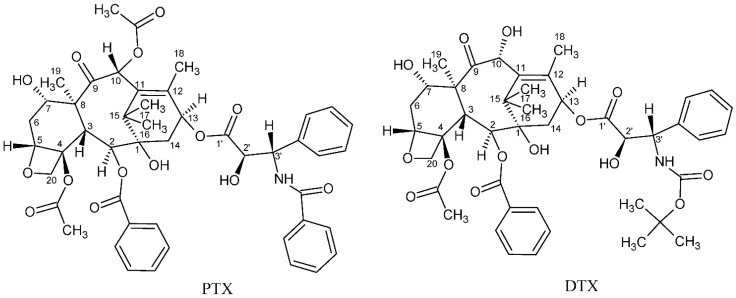
Chemical structures of paclitaxel (PTX) and docetaxel (DTX).

Cancer is considered one of the primary global diseases that lead to morbidity and mortality of millions of people worldwide. Due to its recurrence following chemotherapy treatment, pharmaceutical industries are racing and competing for developing new anticancer agents that have no limitations of adverse reactions or side effects [1,2], aiming to produce more efficient treatment that leads to improved patients’ quality of life [3]. Since nature is considered the best source of drugs, researchers tend to focus more on natural plants and animal products to search for possible cancer cures [4,5,6,7,8,9]. Over the past three decades, taxanes, which are natural diterpenoid substances occurring in yew plants, have been among the most prominent anticancer agents for treating various malignancies. The first taxane to be discovered is Paclitaxel, which was first isolated from the tree bark of the Pacific yew, *Taxus brevifolia* in the sixties of the 20th century. Taxanes are unique hydrophobic diterpenes that have been thoroughly investigated and used to overcome a vast range of cancers including breast, ovarian, lung, prostate, pancreatic, gastric, neck, and head cancer [10]. The naturally derived paclitaxel (PTX, brand name Taxol^®^) and the semi-synthetic analogue docetaxel (DTX, brand name Taxotere^®^) are two taxane drugs used against a number of cancers [11]. Although these two FDA-approved drugs share major similarities in their structures and mechanisms of action, they differ in several other aspects [12]. Their chemical structures are depicted in Figure 1. Taxanes have at least one hydroxyl group that is capable of forming an ester bond with a carboxylic acid. PTX, for example, has three hydroxyl groups to which a carboxylic acid can be attached to form an ester linkage. These hydroxyl groups are located at the C-2’, C-7, and C-1 positions, with their relative order of reactivity generally believed to be C-2’ > C-7 > C-1 (from most reactive to least reactive). DTX is a semi-synthetic analogue of PTX, which was designed due to the scarcity of PTX. It differs from PTX at two positions in its chemical structure. It has a hydroxyl functional group on carbon 10, whereas PTX has an acetate ester group, and it has a *tert*-butyl carbamate ester group on the phenylisoserine side chain instead of the benzyl amide group in PTX (Figure 1). The carbon 10 hydroxyl functional group in DTX makes it more water-soluble than PTX. Water solubility of DTX is 6–7 µg/mL compared to ~0.3 µg/mL of PTX [13].

The primary mode of action of taxanes is to promote and stabilize microtubulin assemblies, preventing their depolymerization and thereby inhibiting the microtubule dynamics that impair mitosis, which leads to cell-cycle arrest and finally to apoptosis [14]. PTX exhibits significant antitumor activity against non-small-cell lung cancer (NSCLC); ovarian, head, and neck tumors; prostate cancer, and breast cancer and is also known as a useful anti-proliferative agent for preventing restenosis [15]. Despite its strong anticancer activity, PTX is poorly water-soluble, exhibits serious dose-limiting toxicities and hypersensitivity reactions (some of which originate from the vehicle cremophorEL^®^, which is required for its formulation) and shows no selectivity for target tissue [16]. Recently, in order to introduce and develop a drug candidate for cancer treatment, we have successfully prepared a novel paclitaxel-lipoate conjugate (IDD-1010) that has an enhanced inhibitory effect on cancer cells, with lower adverse effects [1].

DTX, although it is more water-soluble and is typically formulated in a polyoxyethylene (20) sorbitan monooleate (polysorbate 80): ethanol:saline carrier and administered as an aqueous intravenous solution, still has some side effects, owing to its non-specific targeting behavior [17,18]. As a chemotherapeutic drug, DTX is mainly used for the treatment of breast, ovarian, prostate, and non-small cell lung cancer, but it causes neutropenia, hypersensitivity reactions, peripheral neuropathy, musculoskeletal toxicity, and nasolacrimal duct stenosis [18,19,20,21]. To improve its therapeutic outcomes and evade associated side effects, delivery strategies for DTX have been widely investigated [17,22]. As for treatment of prostate cancer, the prostate specific membrane antigen (PSMA), a transmembrane protein expressed by virtually all prostate cancers, has been suggested to be an important target for DTX treatment [23]. PSMA-targeted small-molecule DTX conjugates were found in in vitro and in vivo studies to have highly increased solubility with less toxic effects [24]. Similarly, the epidermal growth factor receptor (EGFR) that is highly expressed in some prostate adenocarcinomas constituted an important target. Cetuximab, an anti-EGFR monoclonal antibody conjugated to liposomes-containing DTX, showed higher cellular uptake and cytotoxicity as well as enhanced the selectivity of DTX targeting and delivery to prostate cancer cells [25]. In other studies, conjugation of DTX to specific ligands shows increased solubility and targeting [26], controlled-release, and low systemic toxicity [25,27]. Nanoformulation techniques using various nanocarriers have also provided sustained release at adequate concentrations and have thus greatly improved DTX targeting to tumors [17]. Additionally, when co-delivered in combination with other anti-cancer drugs using nanoparticles PEGylation, DTX exhibited enhanced overall efficacy with no apparent toxicity that could be due to better delivery and targeting [17,28]. Nanoparticles in other studies have proven to be beneficial and have made possible enhanced anticancer activity with minimal side effects [17,29]. Despite their promising utility for DTX delivery and targeting, the process and quality control of nanoparticles manufacturing have yet to be optimized. These approaches, whether utilizing nanoparticles or conjugates, demonstrated favorable outcomes manifested by efficient and enhanced DTX higher cellular uptake having increased anticancer potency and leading to reduction in tumor size, with no active DTX-conjugate for prostate cancer yet to show superiority except some variability in their side effects.

**Figure 2:**
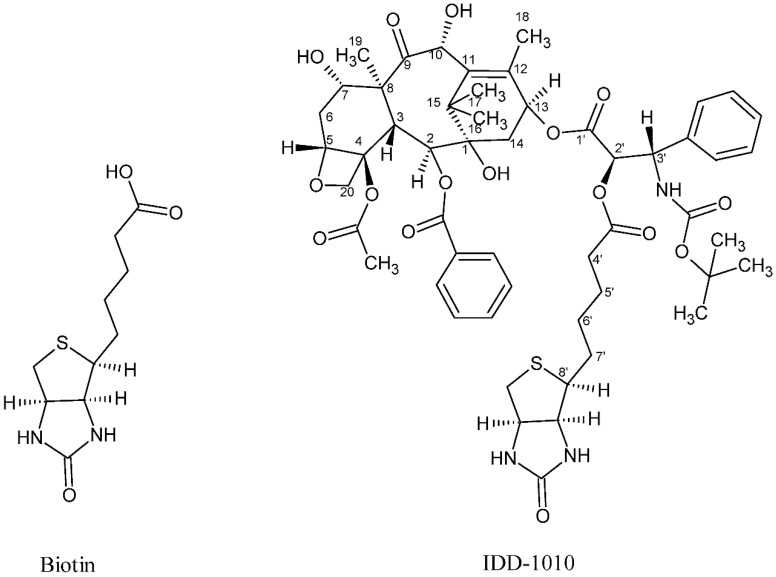
Chemical structure of biotin (left) and the DTX-biotin conjugate (IDD-1010).

In the current study, we introduce a new approach to design and chemically couple biotin to DTX in a single chemical conjugate entity that has an enhanced capability for targeting cancer cells. Biotin, known as vitamin B7, vitamin H, and coenzyme R, is a water-soluble B-complex vitamin that has been linked to DTX because its receptors, which are essential for the uptake of vitamins, are widely distributed on proliferating cancer cells [30]. Biotin is composed of a ureido ring fused with a tetrahydrothiophene ring, and a valeric acid substituent is attached to one of the carbon atoms of the tetrahydrothiophene ring (see Figure 2). As a coenzyme for carboxylase enzymes, which is not produced within the body but supplemented exogenously, biotin plays a major role in the metabolism of carbohydrates, amino acids, and lipids within cells [30,31]. Since proliferating cells require vitamins for their growth, vitamin receptors are typically overexpressed in such cells to enable vitamins to enter [30]. Avidin (or streptavidin) is an example of such a receptor, known for its high binding affinity to biotin [32].

In this work, a novel conjugate of DTX and biotin was designed and successfully prepared to form a new DTX ester. In the conjugate, termed IDD-1010, the biotin is covalently linked to DTX at the carbon atom at position 2’, via an ester bond. The chemical structures of biotin and IDD-1010 are depicted in Figure 2.

The compound IDD-1010 was designed to deliver DTX into proliferating cancer cells. The biotin component is supposed to bind to its cellular receptor, and the DTX is released through hydrolysis of the ester bond with biotin. This hydrolysis can be performed enzymatically by hydrolases or esterases or may simply occur spontaneously in the aqueous environment of diseased tissues, organs, and cells.

## 2. Results and Discussion

### 2.1. Chemical Synthesis

The yield of synthetic IDD-1010 compound was 20.9%, while the purity as assessed by HPLC-PDA was about 98.9% under isocratic conditions (methanol:water-82:18, *v*/*v*) at a flow rate of 1.0 mL/min, with UV detection (at 230 nm) of a 5-μL injection.

### 2.2. +ve-ESi-MS and Elemental Analysis

The molecular formula of the IDD-1010 compound is C_53_H_67_N_3_O_16_S. The calculated exact mass is 1033.4242 Da, while the observed *m*/*z* of protonated [M + H]^+^ is 1034.36 Da. The *m*/*z* peak of 1056.33 Da is indicative of the typical presence of a sodiated adduct of [M + Na]^+^.

The elemental analysis of the theoretical percentages of the atoms and what was observed yielded identical percentages: C, 61.55% (observed: 61.5%); H, 6.53% (observed: 6.53%); N, 4.06% (observed: 4.06%); O, 24.75% (observed: 24.75%); and S, 3.10% (observed: 3.10%).

### 2.3. Biological Study Results

#### 2.3.1. In Vitro Drug Activity

**Figure 3:**
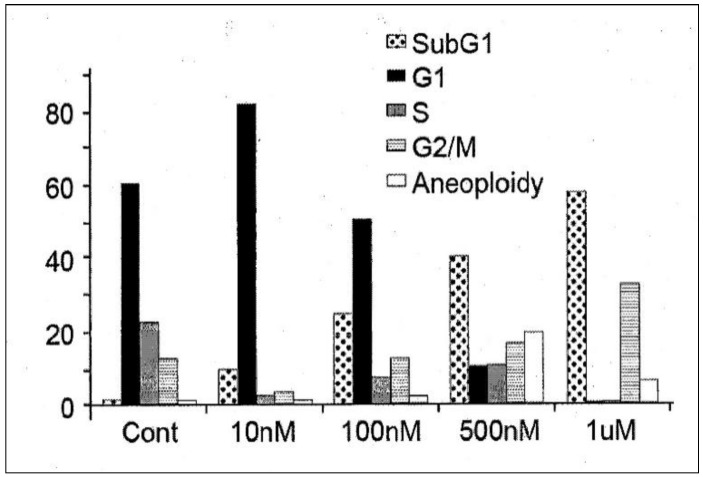
Effects of IDD-1010 on the cell cycle of PC3-Luc cells. PC3-Luc cells were treated with IDD-1010 at the indicated concentrations for 72 h, and were thereafter fixed, stained with propidium iodide, and analyzed by flow cytometry.

In order to determine its efficacy in vitro, IDD-1010 was dissolved in dimethyl sulfoxide (DMSO) and added to PC3-Luc prostate cancer cells at final concentrations of 10 nM, 100 nM, 500 nM, and 1 µM. Seventy-two hours later, the cells were fixed and stained with propidium iodide, and a cell-cycle analysis was performed using flow cytometry. The results are presented in Figure 3; they indicate that IDD-1010 treatment was associated strongly with increased sub-G1 and G2/M populations, which may be indicative of increased apoptosis and mitotic arrest, respectively. As can be seen, the IDD-1010-treated cells show a typical DNA pattern that represents the sub-G1, G1, S, and G2/M phases of the cell cycle. The results show an increase in the cell count in the sub-G1 phase of IDD-1010 treatment, compared to that of the non-treated control cells. The cells treated with 10 nM IDD-1010 show a higher G1 population (83%) compared to 60% in the control. This treatment caused a concomitant increase in the proportion of cells in the G2/M phase of the cell cycle, from the treated PC3-Luc cells with IDD-1010 at concentrations of 10 nM to 1 µM (see Figure 3). Additionally, the percentages of the sub-G1 phase (apoptotic cells) increased significantly after the cells were treated with IDD-1010 at concentrations of 10 nM (an increase of 10%), 100 nM (of 25%), 500 nM (of 40%), and 1 µM (of 58%), compared to 2% in the control (Figure 3). These results suggest that IDD-1010 induces G1-phase cell cycle arrest at a low concentration (10 nM). IDD-1010 treatment at higher concentrations (1 µM) induced cell death in PC3-Luc cells. Aneuploidy, the presence of an abnormal number of chromosomes in cells, has been seen at 500 nM and to a lesser extent at a 1-µM concentration of IDD-1010. The mechanisms by which IDD-1010 inhibits the cell cycle remain to be elucidated.

IDD-1010 at a low concentration (10 nM) inhibited cell proliferation and arrested proliferating PC3-Luc cells in vitro at the G1-phase. However, at higher concentrations (100 nM to 1 µM), IDD-1010 triggered cell arrest at the G2/M phase, and probably apoptosis (Figure 3). Cell cycle arrest at the G2/M phase, occurring through the course of cell division, indicates that the damage to intracellular DNA is difficult to repair and that the cells must undergo apoptosis [33,34]. Since cancer cell proliferation and apoptosis are regulated by cellular signals, the inhibition of these signals is helpful for eliminating cancer [35]. It is worth adding that some other conjugated taxanes were reported recently, such as two novel conjugated derivatives of Chenodeoxycholic acid-Paclitaxel and Ursodeoxycholic acid-Paclitaxel that displayed similar or lesser anticancer activity compared to Paclitaxel [36], and Docetaxel–Palmitate-conjugated derivative [37] that offered a decreased IC_50_ compared to that of Docetaxel.

#### 2.3.2. In Vivo Drug Activity

An orthotopic prostate cancer mice model was utilized to determine the maximum tolerated dose (MTD) of the IDD-1010 compound. The MTD is usually assessed as the maximum dose that can be administered to an animal without compromising its survival by causes other than carcinogenicity. A proper MTD should avoid adverse effects, such as severe adverse behavior and weight loss.

The potential acute toxicity of IDD-1010 was assessed following a single-bolus IV injection, extended over one minute, to male and female Balb/cOlaHsd mice, in order to establish the maximum tolerated dose (MTD). IDD-1010 was injected at four dosage levels of 150, 175, 208, and 250 mg/kg, in a sequential fashion. An additional group of three male and three female mice was injected with the vehicle control item Cremophor” EL:Ethanol Absolute:physiological saline and served as a control group. All surviving animals were observed for a total duration of 14 days. The incidence of mortality was monitored among the animals injected with IDD-1010 at dosage levels higher than 150 mg/kg, amounting to 1/1 female per each dosage level of 175, 208 and 250 mg/kg. Mortality occurred during or immediately following dosing. The mean body weight gain for the 150 mg/kg IDD-1010-treated group at the end of the 14-day study period was not significantly different from that of the control group. No gross pathological findings were noted in any of the animals at the time of their scheduled necropsy, 14 days post-dosing. These results suggest that the MTD level of IDD-1010, when formulated as a stable emulsion in Cremophor” EL:Ethanol Absolute:physiological saline, administered IV as a single-bolus injection over one minute to male or female Balb/cOlaHsd mice, is 150 mg/kg. The MTD for weekly dozing of Docetaxel (Taxotere) as reported by others was observed to be 15 mg/kg in nude mice and 35 mg/kg for FVB/NJ [38].

To determine the efficacy of the compound IDD-1010, an in vivo orthotopic prostate cancer model was generated. Cells of the luciferase-expressing prostate cancer cell line PC3-Luc were injected into the dorsal loop of the mouse prostate. Four weeks after cell injection, drug treatments were initiated and continued for additional three weeks, followed by two weeks of monitoring, as described in the Materials and Methods section. The development of prostate tumors was followed by measurement of the luciferase activity expressed by the injected cells.

#### 2.3.3. Effects of the Different Preparations on Mouse Body Weight

**Figure 4:**
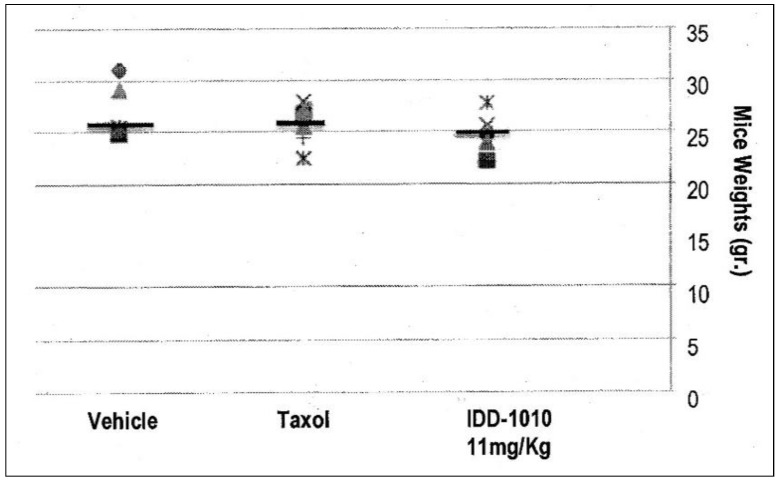
Mouse body weight following injection of IDD-1010, Taxol, and vehicle.

Weight loss in the mice resulted mainly from either drug toxicity or the growth of tumors to a size that exceeded the capability of the body to cope with. To test the effect of IDD-1010 on their weight, the surviving animals were weighed every week and at the end of the experiment. The recorded data are presented in Figure 4, which shows that IDD-1010 treatment did not lead to any significant weight loss; the *p*-value, in comparison to the vehicle group’s, was 0.1837. The average weight of the vehicle group was 27 g.

Mouse body weight was recorded every week and on week eight following cancer cell injection. The black lines represent the median. The *p*-values for the Taxol and IDD-1010 groups in comparison to vehicle are >0.05, i.e., 0.3768 and 0.1837, respectively.

#### 2.3.4. Tumor Volume Kinetics

**Figure 5:**
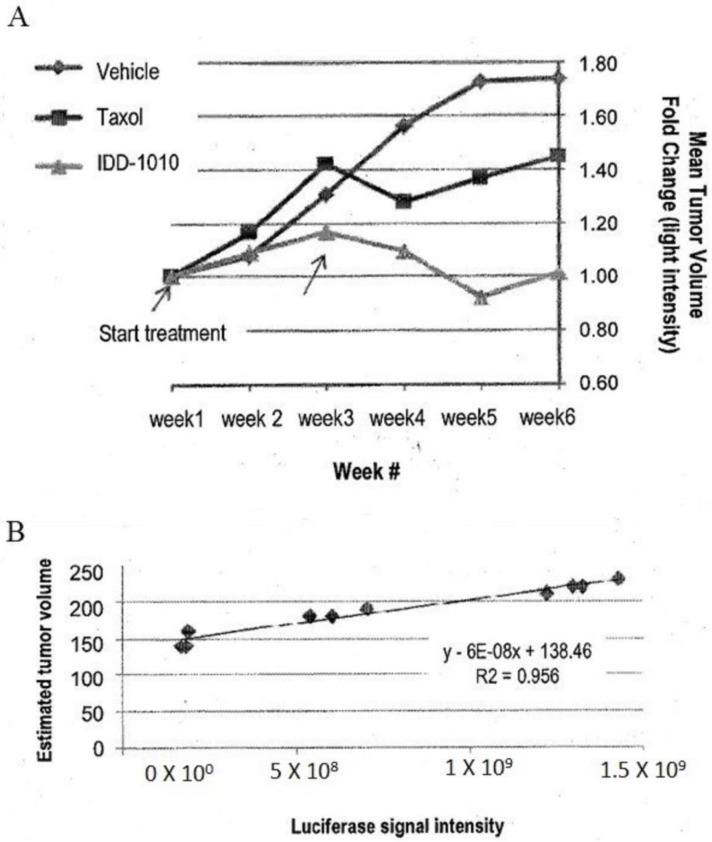
Prostate tumor (PC3-Luc) volume fold change based on luciferase signal intensity (**A**) and tumor volume estimation curve based on luciferase signal intensity of some tumors against their tumor volume at the beginning of the experiment obtained from the three mice that were sacrificed before the start of treatment (**B**).

To estimate the starting tumor volume and to follow the growth rate of the tumors throughout the study, three mice were sacrificed before the initiation of treatment and were measured for their tumor volume and luciferase activity. From these data, a correlation curve was established, in order to estimate tumor volume at any time throughout the experiment, based on the following equation:

Estimated tumor volume = 6 × 10*^b^ X* + 138.46

where *X* equals the luciferase intensity reading (see Figure 5B).

The estimated tumor volume at the start of drug injection was about 200 mm^3^. The data are presented in Figure 5. As can be seen there, the relative luciferase activity values show that IDD-1010 was more potent than Taxol and was the only treatment that led to a decrease in tumor volume.

#### 2.3.5. Tumor Volume

**Figure 6:**
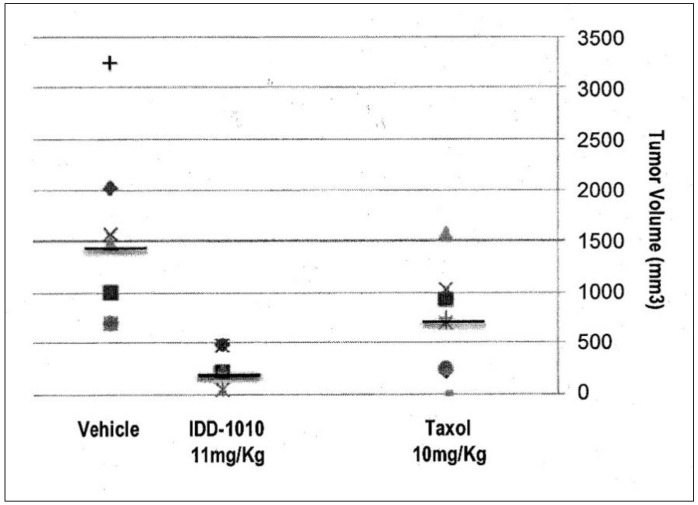
Prostate tumor (PC3-Luc) volume following treatment with IDD-1010, Taxol, and vehicle. By the end of the experiment, the tumor volume in each treatment group was determined by measuring the short and the long dimensions of the tumor and calculating volume according to the following equation: Volume = [(the shortest diameter)^2^ × (the longest diameter)]/2. The black lines represent median values.

To determine the anti-cancer activity of IDD-1010, tumor volumes at the end of the experiment were measured, and the average tumor volume for each treatment group was calculated. The data for this are presented in Figure 6; they show that Taxol and IDD-1010 caused a statistically significant reduction in tumor volume compared to the vehicle group, with final average tumor volumes of 681 and 289.4 mm^3^, respectively, compared to 1737 mm^3^ in the vehicle group.

#### 2.3.6. Tumor Weight

The effect of the compounds on tumor development was also assessed by measuring tumor weight at the end of the experiment. Taxol and IDD-1010 treatment resulted in a statistically significant reduction in tumor weight compared to the vehicle group (data not shown). The *p*-values in comparison to the vehicle were 0.0192 and 0.0011 for Taxol and IDD-1010, respectively. It has been suggested that inconsistencies between tumor volume and tumor weight might result from the fact that the measurement of tumor volume is inaccurate because most tumors have irregular architecture.

#### 2.3.7. Mice Survival

**Figure 7:**
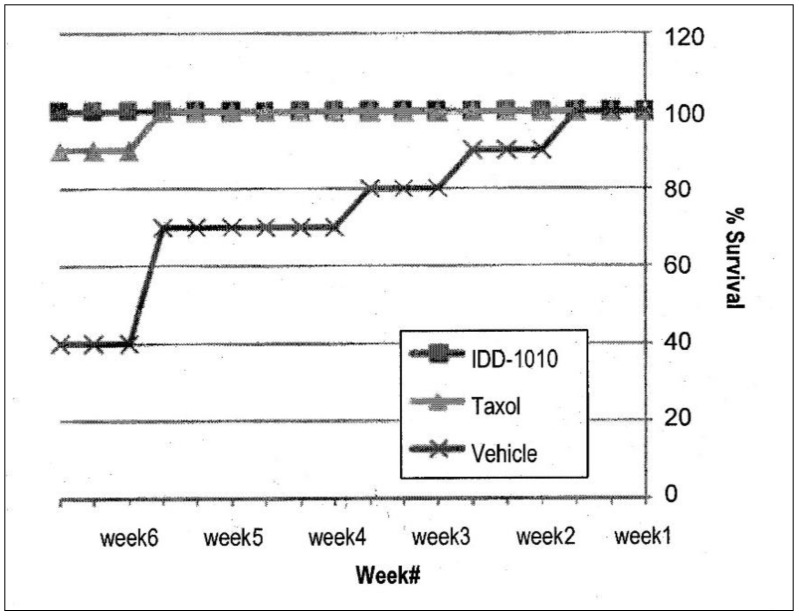
Animal survival and mortality curves following the administration of IDD-1010, Taxol, and vehicle. The mice were treated once a week for three constitutive weeks with a total of four injections. The comparison plot shows the percentages of mice survival, determined by counting the dead mice in each treatment group throughout the experiment.

To determine the efficacy of treatment, mice survival was also observed. Using the Kaplan-Meier estimator, the numbers of animals dying throughout the experiment were plotted, and the percentages of the different groups were compared. The results are presented in Figure 7; they show that the Taxol and IDD-1010 groups both had a 100% survival rate, whereas in the vehicle group, where tumor growth was unimpeded, a 40% survival rate was observed.

## 3. Materials and Methods

Biotin, PTX, DTX, DCC, DMAP, dry dichloromethane, diethyl ether, and dichloromethane were all purchased from Sigma (Rehovot, Israel). HPLC grade acetonitrile (ACN) and ultrapure water were purchased from Biolab (Jerusalem, Israel).

### 3.1. Purity of IDD-1010

The purity of IDD-1010 was determined using an HPLC system (Thermo Scientific, San Jose, CA, USA), which included an Accela Pump with a degasser module, an Accela autosampler, and an Accela PDA detector. An HPLC-cartridge LiChroCART^®^ 250-4 LiChrospher^®^ 100 RP-18 (5 μm) (Merck, Roch-Ha’ayin, Israel) guard column and a LiChroCART^®^ 4-4 LiChrospher^®^ 100 RP-18 (5 μm) guard column were used to protect the analytical column.

### 3.2. Chemical Characterization

The chemical structure of IDD-1010 was confirmed by mass spectrometry (MS) measurements in the positive ESI mode, along with the elemental analysis data obtained for this product.

#### 3.2.1. (+)-ESi-MS

The molecular mass was determined by (+)-ESI-MS (electrospray ionization mass spectrometry) in positive mode (using an LTQ Orbitrap XL hybrid ion trap with a high-resolution Orbitrap detection system (Thermo Scientific, Waltham, MA, USA)).

#### 3.2.2. Elemental Analysis

Determination of C, H, and N was performed with a Perkin-Elmer 2400 Series II Analyzer, which uses a combustion method (950–1000 °C) to convert the sample elements to simple gases. The system uses a steady-state, wave front, chromatographic approach to separate the controlled gases, which are detected through their thermal conductivity. High-speed microprocessor control, solid-state components, and built-in diagnostics ensured good performance and reliability. The measurement of sulfur percentage was done with the oxygen-flask combustion method and subsequent gravimetric titration through ion chromatography analysis, using a Dionex IC system.

### 3.3. Synthesis of IDD-1010

**Figure 8:**
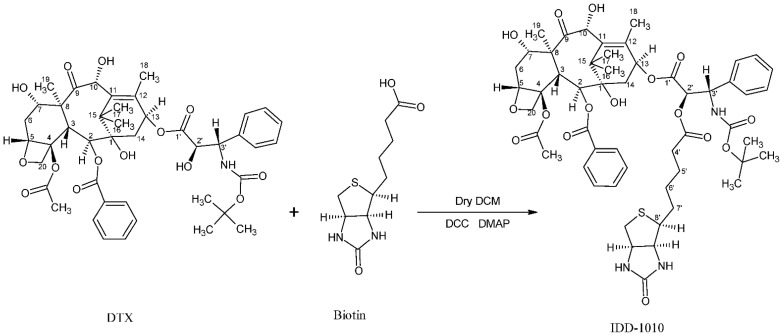
The synthesis pathway of IDD-1010.

Biotin (36 mg; 0.00015 mol), 4-dimethy1 amino pyridine (DMAP), and *N*,*N*′-dicyclohexylcarbodiimide (DCC) (0.029 g; 1 eq) were added to a solution of DTX (100 mg) in dry dichloromethane (10 mL) under nitrogen gas, and the reaction mixture was stirred at room temperature for 24 h (Figure 8). Then the mixture was diluted with diethyl ether, and the docetaxel-biotin conjugate (i.e., IDD-1010) precipitated from it. The precipitate was further purified by column chromatography to afford a product at a yield of about 20.9% and a purity, as measured by HPLC at 230 nm, of about 98.9%. The product was further characterized by positive ESI-MS and elemental analysis.

### 3.4. Drug Candidate Formulation for the In-Vivo Experiments

#### 3.4.1. Preparation of IDD-1010 Dosing Solutions

To prepare the 1-mL emulsions at the required concentrations, on each day of dosing, the appropriate amount of IDD-1010 powder was weighed and dissolved in 175 µL of cremophor@EL:ethanol absolute (80%/20% *v*/*v*; 140 µL of cremophor@EL, and 35 µL of ethanol). The solution was then mixed until the IDD-1010 powder was completely dissolved. Subsequently, the solution was diluted with 825 µL of physiological saline to obtain 1 mL of stable emulsion at the appropriate concentration. Different volumes of IDD-1010 emulsions are prepared in an identical fashion, and the ratios among all components are maintained.

#### 3.4.2. Preparation of the Vehicle Control

On the day of dosing, 140 µL of Cremophor@EL was mixed with 35 µL of ethanol absolute (80% to:20% *v*/*v*). The solution was then diluted with 825 µL of physiological saline. Different volumes of vehicle control are prepared in an identical fashion as long as the ratios between all components are maintained.

### 3.5. Animals

Young adult (male and female) Balb/cOlaHsd mice, eight to nine weeks old at the start of the study, (obtained from Harlan Laboratories Israel, Ltd. (ISO 9001:2008 Certificate No.: GB06/68708) (Tel Aviv, Israel) were used. The weight variation of animals at the time of treatment initiation did not exceed +20 % of the mean weight for each sex. Healthy mice were acclimated for at least five days. The animals were housed within a limited-access rodent facility and kept in same-sex groups of three in polypropylene cages fitted with solid bottoms and filled with wood shavings as bedding material. The environment was controlled automatically by computer and set to maintain the temperature at 20–24 °C, with a relative humidity of 30–70%, a 12-h light/dark cycle, and 10–30 air changes/hour in the study room. The animals were fed with certified commercial rodent diet ad libitum, with free access to drinking water, supplied to each cage via polyethylene bottles with stainless steel sipper tubes. The water was filtered (0.1 q filter), chlorinated, and acidified. Each animal was given a unique identification ear number, which also appeared on a cage card posted on the front of each cage. The cage card also contained the study number, the route of administration, the sex and strain of the occupant, and all other details pertaining to the treatment group and dose levels. On the day of arrival, the animals were randomly assigned to the various test groups. At the end of the experiment, the surviving animals were euthanized by COC asphyxiation prior to necropsy. Animals euthanized for humane reasons were sacrificed by the same method. The mice were divided into five groups of six (each group containing three animals of each sex). Four of the groups received three appropriately spaced doses of IDD-1010. The other group received vehicle only. The initial target dosage level was 150 mg/kg. Additional appropriate dosage levels were determined during the course of the study, based on the reactions observed. The volume of the injections was 10 mL/kg.

### 3.6. Study Design

This study was aimed at determining the acute toxicity (MTD) of IDD-1010 using a stepwise procedure with a minimal number of animals per step. An initial single target dose of 150 mg/kg was injected IV into one group of three male and three female mice. Depending on the observed presence or absence of lethality or severe adverse reactions to treatment, further groups were then injected with either higher or lower appropriately spaced doses. Administration was sequential, and the intervals between doses were determined according to the onset, duration, and severity of toxic signs; they were delayed until it was fairly certain that the dosed animals would survive. An additional group of mice was injected with the vehicle at an equal volume and dosage and served as a control group. All of the dosed animals that survived were observed for 14 days, after which they were sacrificed and underwent gross necropsy. The IDD-1010 formulation and the vehicle control formulation were injected into a tail vein by intravenous slow bolus injection over approximately one minute, with a suitable syringe and needle, after warming the cage environment for 3–5 min and/or warming the tail vein using a gauze pad that had been soaked in warm water.

### 3.7. In Vitro Drug Activity

PC3-Luc prostate carcinoma cells (5.0 × 10^5^) were cultured in 60-mm plates in triplicate. One day later, the cells were treated with several different concentrations of IDD-1010 (10 ng/mL to 50 µg/mL). Seventy-two hours post-treatment, the cells were collected, fixed, and stained with propidium iodide. To determine the efficacy of IDD-1010, cell cycle analysis was performed to determine the fraction of cells at each stage of the cell cycle.

### 3.8. Prostate Orthotopic Injection of PC3-Luc Cells

PC3-Luc cells (0.5 × 10^6^) were injected into the prostate of six-week-old male NOD/SCID mice. The mice were imaged weekly for eight weeks to monitor tumor growth kinetics. At the end of this time, the mice were euthanized, and their tumors were excised, weighed, and preserved for further histopathological analyses.

### 3.9. Preparation of Tumor Cells

PC3-Luc cells (maintained in RPMI, 10% fetal bovine serum (FBS) and supplements) were trypsinized, returned to the same growth medium, and re-suspended to achieve a concentration of 0.5 × 10^6^ cells in 20 µL of solution.

### 3.10. Injection

Six-week-old male NOD/SCID mice were anesthetized with an IM injection of ketamine (120 mg/kg)/xy1azine (6 mg/kg). Once anesthetized, the mice were placed in a supine position, and the surgical area was sterilized three times with alternate wipes of 70% ethanol and iodine. A 5–10 mm incision along the posterior midline of the abdomen was made to expose the prostate just beneath. Another incision was made in the peritoneal wall. The bladder was retracted and pressed lightly to expose the prostate. PC3-Luc cells (0.5 × 10^6^ cells in 20 µL) were slowly injected into the dorsal prostatic lobe, using a 30-gauge needle attached to a Hamilton syringe. A well-localized bleb within the injected prostatic lobe indicated a technically satisfactory injection. The peritoneal incision was sutured using 6-0 silk suture with a C-22 reverse cutting needle, and the skin was closed with two or three autoclips. During recovery, the mice were given petroleum ointment over the eyes and 500 µL of warm PBS that was injected subcutaneously on the back. Once they recovered, the mice were injected subcutaneously with 50 q1 of buprenorphine (0.05–0.1 mg/kg) and returned to their cages.

### 3.11. Imaging

The mice were injected with 100 µL of luciferin (15 mg/mL stock) per 10 g of mouse body weight by IP injection in the animal’s lower left abdominal quadrant. After 8–10 min, they were anesthetized with gas anesthesia (3% isoflurane). Once anesthetized (for two to three minutes), they were placed on black paper and imaged ventrally. They were imaged for the first time on the seventh day after their wounds had healed, to ascertain the successful implantation of tumor cells, and then once a week for eight weeks. At the end of the experiment, the animals were euthanized, and tumors and selected tissues were assessed by histopathological analysis.

### 3.12. Drug Treatment

After it had been ascertained that the injections were successful and that the tumors were well established (with a tumor volume of about 200 mm^3^), the mice were divided randomly into two groups. IDD-1010 was dissolved completely in a 175-µL solution of cremophor:ethanol 80%:20% *v*/*v*. Then, 825 µL of saline was added dropwise and mixed to formulate a 1-mL solution. On days 28, 35, 42, and 49 after the cell injections, the groups were injected IV with the IDD-1010 solution at a concentration of 11 mg/kg of mouse body weight. There was a total of four injections, the first given on day one and the three others once a week for three sequential weeks, followed by two weeks of monitoring.

### 3.13. Clinical Observations

Individual clinical examinations were carried out immediately post-dosing, up to the first 30 min and at least twice more during the first two hours. Thereafter, the animals were observed at least once daily, or more frequently when indicated by the response to treatment, for a total duration of 14 days (on weekends, cage-side observations were performed). The observations encompassed changes in the skin, fur, eyes, and mucous membranes; the occurrence of secretions and excretions (e.g., diarrhea); and autonomic activity (e.g., lacrimation, salivation, piloerection, unusual respiratory patterns). Additionally observed and recorded were posture, changes in gait, responses to handling, and occurrences of bizarre behavior, tremors, convulsions, sleep, and coma. Any local reactions at injection sites were recorded as well. All of the animals were checked for morbidity and mortality, twice daily on weekdays and once daily on weekends and official holidays

### 3.14. Body Weight

The body weight of all the animals was taken upon their arrival, shortly before dosing, on the second and seventh days post-dosing, and prior to termination (14 days post-dosing). In the case of decedents, body weight was measured as close as possible to the time of death (when applicable).

### 3.15. IDD-1010 Activity In Vivo Xenograft Model

A total of 2 × 10^6^ A549 human non-small-cell lung carcinoma cells, suspended in 100 µL of PBS (grown and supplied by PharmaSeed Ltd., Ness Ziona, Israel) were injected SC into the dorsal side/ flank of each of the 60 NOD-SCID female mice, aged six to eight weeks, with an average weight of 20–25 g. The body weight of the animals was measured before the commencement of the study, once a week throughout treatment for the duration of the study, and before they were sacrificed. The data were recorded in an Excel file. Tumor volume was monitored with calipers; the length and width of each tumor was measured twice weekly from the beginning of the study, throughout treatment, up to the end of the study. Tumor volume was calculated, and the data were recorded in an Excel file. When tumor volume reached 70 to 100 mm^3^, the mice were randomly divided into three groups with five or six in each group, and treatment was started. The tumor-bearing mice were injected IV in the tail vein via slow injection for two minutes with the appropriate solutions, as indicated in the following schedule.

Group 1 (five mice): Vehicle, dose: 10 mL/kg, unless otherwise stated.

Schedule: Four injections, given once a week for three consecutive weeks with the first on day one. At the end of treatment, the mice were monitored for two additional weeks.

Group 2 (five mice): Docetaxel, dose: 7.4 mg/kg. Schedule: A total of four injections, the first was given on day one and the three others once a week for three sequential weeks. At the end of treatment, the mice were monitored for two additional weeks.

Group 3 (nine mice): IDD-1010, dose 10 mg/kg. A total of four injections, the first was given on day one and the three others once a week for three sequential weeks. At the end of treatment, the mice were monitored for two additional weeks.

The observations of clinical signs and survival were done within the immediate cell line injection, and examinations and drug injection intervals were adjusted periodically when the signs warranted, over the course of the entire experiment. The data were recorded in an Excel worksheet. At the termination of the experiment, the whole tumor mass was harvested, weighed, sized with calipers, calculated, photographed with camera, and kept in a formaldehyde solution. Vital organs were preserved in a 4% formaldehyde solution for the purposes of histology and organ lysate preparation. A macroscopic evaluation of inner organs was performed to detect any abnormalities or metastases. All of the data were recorded. The results show an inhibition of tumor growth in Group 3.

### 3.16. Statistical Analysis

The data were analyzed using the data analysis module in Excel (v16.0, Microsoft, Redmond, WA, USA). The differences between the control (untreated) and treatment groups were evaluated by applying a one-way analysis of variance (ANOVA). A *p*-value of less than 0.05 was considered statistically significant. The data shown in the figures display the means and standard deviations.

## 4. Conclusions

Despite the fact that taxanes (Paclitxel, Docetaxel, and Cabazitaxel) have broad activity, their severe toxicity [20,21,39,40] limits the treatment dose and thereby narrows the therapeutic window [39,40]. Overexposure to taxanes results in severe toxicity and side effects and leads to early discontinuation of treatment. Therefore, circumventing the occurrence of taxanes side effects has attracted interest to the nanoformulation of the drug and its use in combination with other drugs [17,41]. Naturally occurring molecules with chemopreventive and chemotherapeutic properties have been used to construct model molecules for the development and validation of new drug candidates [42]. We have approached this problem herein, aiming at widening the therapeutic window of docetaxel by chemically coupling it to biotin and thereby producing a new conjugate molecule, IDD-1010. We conclude from the current study that the anti-tumor activity of IDD-1010 is far more favorable than that of natural taxane (approved drug). IDD-1010 has a wider therapeutic window with a higher maximum tolerated dose and superior tumor inhibition. Its efficacy is evidenced in this study by a decrease in the volume and weight of tumors, as well as by a lower mortality rate in the mouse test groups. All of the results demonstrate that IDD-1010 has great potential as a new drug for cancer treatment, and hence, it is recommended for advancement into clinical trials.

## Sample Availability

Samples of the compounds are available from the authors.
